# NDVI changes in the Arctic: Functional significance in the moist acidic tundra of Northern Alaska

**DOI:** 10.1371/journal.pone.0285030

**Published:** 2023-04-28

**Authors:** R. Gus Jespersen, M. Anderson-Smith, P. F. Sullivan, R. J. Dial, J. M. Welker

**Affiliations:** 1 Department of Biological Sciences, University of Alaska Anchorage, Anchorage, Alaska, United States of America; 2 Anchorage, Alaska, United States of America; 3 Environment and Natural Resources Institute, University of Alaska Anchorage, Anchorage, Alaska, United States of America; 4 Alaska Pacific University, Anchorage, Alaska, United States of America; 5 Ecology and Genetics Research Unit, University of Oulu, Oulu, Finland; 6 University of the Arctic (UArctic), Rovaniemi, Finland; Michigan State University, UNITED STATES

## Abstract

The Normalized Difference Vegetation Index (NDVI), derived from reflected visible and infrared radiation, has been critical to understanding change across the Arctic, but relatively few ground truthing efforts have directly linked NDVI to structural and functional properties of Arctic tundra ecosystems. To improve the interpretation of changing NDVI within moist acidic tundra (MAT), a common Arctic ecosystem, we coupled measurements of NDVI, vegetation structure, and CO_2_ flux in seventy MAT plots, chosen to represent the full range of typical MAT vegetation conditions, over two growing seasons. Light-saturated photosynthesis, ecosystem respiration, and net ecosystem CO_2_ exchange were well predicted by NDVI, but not by vertically-projected leaf area, our nondestructive proxy for leaf area index (LAI). Further, our data indicate that NDVI in this ecosystem is driven primarily by the biochemical properties of the canopy leaves of the dominant plant functional types, rather than purely the amount of leaf area; NDVI was more strongly correlated with top cover and repeated cover of deciduous shrubs than other plant functional types, a finding supported by our data from separate “monotypic” plots. In these pure stands of a plant functional type, deciduous shrubs exhibited higher NDVI than any other plant functional type. Likewise, leaves from the two most common deciduous shrubs, *Betula nana* and *Salix pulchra*, exhibited higher leaf-level NDVI than those from the codominant graminoid, *Eriophorum vaginatum*. Our findings suggest that recent increases in NDVI in MAT in the North American Arctic are largely driven by expanding deciduous shrub canopies, with substantial implications for MAT ecosystem function, especially net carbon uptake.

## Introduction

Because large swaths of the Arctic are remote, satellites equipped with sensors that quantify reflected visible and infrared radiation at large scales are critical to understanding the effects of high-latitude climate change [1–5]. In particular, NDVI has frequently been used to estimate vegetation biomass, leaf area, or species composition [6–9], often with the goal of predicting ecosystem functional attributes, such as photosynthetic and respiratory fluxes of CO_2_ [9–12]. However, accurate interpretation of the NDVI signal in the Arctic remains limited by relatively few ground truthing efforts [7,13], hindering our ability to understand the causes and functional consequences of contemporary NDVI trends [14].

NDVI has increased (“greening”) across large portions of the Arctic since the 1980’s [15–19]. This trend is spatially heterogeneous, and has in some areas slowed, with other areas showing declining NDVI (“browning”) [20,21]. However, the higher NDVI of deciduous shrub tundra relative to other tundra types [22,23] has led many researchers to suggest that increasing NDVI indicates a widespread increase of deciduous shrubs in the Arctic [2,3]. High-resolution photographs and field validation have confirmed the linkage between increasing Landsat NDVI and expanding shrub cover in some parts of the Arctic [7,13,18,23–26]. However, questions remain regarding how the Arctic NDVI signal, beyond indicating shifts in vegetation community composition, relates to ecosystem structure and function.

In several cases the relationship between NDVI and LAI has been used to extend the utility of NDVI to metrics of ecosystem function, including estimates of gross primary productivity (GPP) and net ecosystem CO_2_ exchange (NEE) [10,11]. Often in such efforts, ground truth plots are sampled across a range of vegetation communities or experimental treatments, and in this context NDVI has frequently demonstrated a strong relationship with LAI or total biomass and, subsequently, CO_2_ uptake [6,9–12]. However, when sampled *within* the more productive vegetation types that are critical to the Arctic carbon (C) cycle it remains to be demonstrated whether spatial or temporal variation in NDVI indicates changes in leaf area and/or deciduous shrub abundance. For example, moist acidic tussock tundra (MAT) is a widespread, highly productive Arctic ecosystem which holds globally important stocks of soil C [27]. Within MAT, peak-summer LAI values often approach or exceed an LAI of 1.0 [13,28,29]; above this threshold, the observed NDVI-LAI relationship may be confounded, as higher LAI values will typically coincide with greater canopy layering.

The goal of this project was to use *in situ* measurements of vegetation structure and ecosystem function to improve interpretation of changing NDVI within MAT. We aimed to answer two key questions. First, does increasing NDVI in MAT indicate an increase in leaf area, deciduous shrub abundance, or both? And second, what are the implications of observed variation in NDVI for NEE, GPP, and ecosystem respiration (*R*_*eco*_)? To answer these questions, we coupled measurements of NDVI in 70 MAT plots (0.49 m^2^) with NDVI measurements from 199 homogeneous plots (0.03 m^2^) of MAT cover types and 89 leaves of the three dominant species. We combined this with measurements of ecosystem CO_2_ exchange in the MAT plots over two growing seasons. We discuss our findings in the context of models commonly used in predicting pan-Arctic NEE and take the additional step of parameterizing and evaluating two of these models with our intra-MAT dataset.

## Methods

### Study site

This study was conducted primarily during the summers of 2010 and 2011 at Toolik Lake Field Station (68°38’N, 149°36’W, elevation 720 m) and within the Imnavait Creek watershed 10 km north of Toolik Lake in northern Alaska (S1 Fig). Permits for the work were provided by Toolik Lake Field Station and the Bureau of Land Management. The broader study area is in the northern foothills of the Brooks Range and is characterized by gentle, rolling topography. Average annual air temperature is about -7° C, mean annual precipitation is 322 mm, and the area is snow-covered approximately 8 months of the year with a mean growing season length of 125 days [30].

MAT in this region is dominated by the tussock forming sedge, *Eriophorum vaginatum*, deciduous dwarf shrubs (primarily *Betula nana* (dwarf birch) and *Salix pulchra* (diamond-leaf willow)), and dwarf evergreen shrubs (mainly *Vaccinium vitis-idaea* (lingonberry) and *Ledum palustre* (Labrador tea)). The soil surface is composed primarily of moss and litter.

### Plot selection

To provide inference across a broad range of potential MAT vegetation conditions, 70 sample plots (70 x 70 cm) were established in MAT areas that demonstrated a wide range of deciduous shrub and graminoid cover. Fifty-three plots were sampled near Toolik Lake and seventeen near Imnavait Creek.

### Species cover and leaf area

We estimated species composition in our plots using the point-frame technique with 100 evenly spaced points [31] in a 0.49 m^2^ frame. The pin, a 3 mm diameter x 1 m long rod, was lowered at each intersection, and each contact with plant or litter was recorded. We itemized 13 species of vascular plants as well as moss, lichen, litter, and standing dead material (leaves and stems), for a total of 17 categories. The ‘top cover’ of each species was calculated as the proportion of first pin hits whereas ‘repeated cover’ was calculated as the proportion of all pin hits [32]. Vertically projected leaf area (LA_VP_) of each vascular plant species was calculated as the sum of all pin hits attributed to that species’ leaves divided by 100 (the maximum number of hits in a single layer); this served as a non-destructive estimator of plot-level LAI. Pin intercept methods for LAI estimation have been shown to be highly correlated with harvest-based LAI, typically explaining 80–90% of the variation [33,34]. LA_VP_ would equal LAI if all leaves were arranged perpendicular to the pin [32]. However, accounting for variation in leaf orientation is challenging and was not attempted here. Plot-level LA_VP_ was calculated in the same manner as species-level but with leaf pin hits summed across all vascular plant species. We also calculated LA_VP_ with all moss pin hits included and discuss the implications of this using this version. Top and repeated cover and LA_VP_ were later grouped into eight cover types (CTs): deciduous shrub, evergreen shrub, graminoid, forb, lichen, litter, moss, and standing dead material. The live CTs are henceforth referred to as plant functional types (PFTs) and represent groupings commonly used in Alaskan vegetation studies [26,35]. Species groupings can be viewed in Fig 2.

### Spectral reflectance measurements

A dual channel portable spectroradiometer (Unispec-DC, PP Systems International, Inc., Amesbury, MA) was used to measure plot-level reflectance, usually at the same time as the flux measurements and in all cases within three hours of solar noon and within two days of measuring plot CO_2_ exchange. The upward looking foreoptic of the spectroradiometer, covered by a diffuser, measures hemispheric irradiance of the sky. The downward looking foreoptic measures conic radiance of the vegetation and has a field of view that is approximately 20°; this was placed *h* = 199 cm above plot center such that the radius of the field of view was *r* = 35 cm (given that tan 10° = *r*/*h* and tan 10° = 0.1763). The two channels of the spectroradiometer with their attached foreoptics were calibrated according to manufacturer recommendations using a small, highly reflective Spectralon disk (Labsphere, Inc., North Sutton, NH). Plot radiance and sky irradiance were simultaneously logged by the Unispec-DC. NDVI was calculated using average reflectance in the red (620 nm to 670 nm), and near infrared (841 nm to 876 nm) spectral regions, which correspond to MODIS bands 1, and 2, respectively, using the formula:

$$\mathrm{NDVI}=\left(R_{nir}-R_{red}\right)/\left(R_{nir}+R_{red}\right)$$

where *R*_*nir*_ and *R*_*red*_ are the average reflectance in the near infrared and red bands, respectively. We measured reflectance on our plots at approximately two-week intervals, beginning on June 24 and ending on August 14.

To better understand the contribution of different CTs to the spectral signature of MAT, we measured reflectance of the dominant CTs two ways: as monotypic stands of important CTs and as individual leaves of *B*. *nana*, *S*. *pulchra*, and *E*. *vaginatum*. For 199 naturally occurring monotypic stands at least 20 cm in diameter (20 graminoid, 94 deciduous shrub, 31 evergreen shrub, 21 moss, 4 standing dead material in the canopy, and 29 litter) we measured reflectance using the same technique as in the flux plots (but with the downward looking foreoptic fixed at 57 cm height for a 20 cm diameter field of view). Selecting only patches of pure composition resulted in uneven sample sizes, but the two most abundant and ecologically important PFTs (deciduous shrubs and graminoids) were well represented. For individual leaves of *B*. *nana*, *S*. *pulchra*, and *E*. *vaginatum* (n = 31, 31, 27, respectively) we measured reflectance spectra in the field with a Unispec-DC on June 28 through July 3, 2013. A device was constructed to hold the leaves exactly 1.7 cm below the downward looking optic so the diameter of the field of view was 1 cm (S1 Fig). Leaves were collected and immediately fastened to black blocks with black plastic tape and scanned in the field. Leaves *of B*. *nana* and *S*. *pulchra* are generally more than 1 cm wide so only one leaf was used for each scan. Ten or more green *E*. *vaginatum* leaves were placed taped together such that they lay parallel with zero space between blades and were scanned at one time. Each sample was scanned in three times, rotating 90° between scans (but always remaining perpendicular to the foreoptic), and the scans were averaged to produce a single NDVI value for each sample.

### CO_2_ exchange measurements

We measured plot-level CO_2_ exchange (NEE, GPP, and ER) in an enclosed clear acrylic chamber (70 cm square by 40 cm high, with ~.0.9 cm thick walls) using a LI-6400 portable photosynthesis system (LI-COR Environmental, Lincoln, Nebraska) attached to the side of the chamber. The chamber contained two small 2.4 W fans and a LI-190 quantum sensor (LI-COR Environmental, Lincoln, Nebraska) and was sealed against a plastic base frame fitted with a 50 cm-wide flexible vinyl skirt, which was sealed to the tundra with a heavy (~15 kg) chain. After sealing the chamber, we measured chamber CO_2_ concentration every two seconds for one minute. We calculated NEE according to:

$$\mathrm{NEE}=\frac{\rho V}{A}\frac{dC}{dt}$$ (1)

where NEE is net CO_2_ flux (μmol m^-2^ s^-1^), *ρ* is the average molar density of air during the measurement interval (mol m^-3^), *V* is the volume of the chamber (m^3^), *dC/dt* is the change in CO_2_ concentration over time (μmol CO_2_ mol^-1^ s^-1^), and *A* is the footprint area of the chamber (m^2^). At each plot we measured NEE under six light levels (implemented with shade cloths), ventilating the chamber between light levels by tipping it upright for 15–20 seconds until the CO_2_ concentration returned to ambient. The resulting dataset included 2386 individual flux measurements. Mean ± SD *R*^*2*^ values for the linear fits used to calculate NEE from the raw data were 0.92 ± 0.11.

NEE was modeled as rectangular hyperbola mixed-effects model using the nlme package [36] in R, with both plot and day of year as random effects:

$$\mathrm{NEE}=R_{eco}-\frac{A_{max}\mathrm{PAR}}{k+\mathrm{PAR}}$$ (2)

where NEE is the net flux of CO_2_ measured in the chamber (μmol m^-2^ s^-1^), *R*_*eco*_ is modeled ecosystem respiration (μmol m^-2^ s^-1^), *A*_*max*_ is a modeled parameter indicating maximum assimilation rate of CO_2_ (μmol m^-2^ s^-1^), PAR is incident flux of photosynthetically active radiation measured in the chamber as (μmol photons m^-2^ s^-1^), and the modeled parameter *k* is the PAR value at half *Amax* (μmol photons m^-2^ s^-1^). Starting values for these parameters in the nlme estimation procedure were: *R*_*eco*_: NEE measured under full shade, *k*: PAR under full light * 0.5, *Amax*: NEE measured under full light. The nlme model predicted observed values well (*R*^*2*^ = 0.95, predicted vs. observed slope = 0.93). To minimize the effect of measurement error during the full shade measurement and for consistency because the other CO2 flux variables used in our subsequent analyses are also fitted values, the *R*_*eco*_ parameter estimated from the light curve fits was used in further calculations (See S2 Fig for comparison). As expected, *k* and *A*_*max*_ displayed a logarithmic relationship and *A*_*max*_ and *k* followed the seasonal patterns of increase and decrease typical for the Arctic (S3 Fig). NEE was calculated at a light level typical of this area during midsummer (PAR = 600 μmol photons m^-2^s^-1^). These values were then used to calculate GPP_600_ as the sum of NEE_600_ and *R*_*eco*_. The plot-level light compensation point (LCP), where NEE = 0 (when *R*_*eco*_ *=* GPP), was determined from the x-intercept of the fitted light curve for those curves with sufficient GPP to drive NEE below zero. The initial slope of the light curve at PAR = 0, commonly referred to as quantum yield (*E*_*o*_), was calculated by taking the first derivative of the light curve equation with respect to PAR and setting PAR = 0, yielding:

$$E_{0}=-\frac{A_{max}*k}{({k)}^{2}}$$ (3)

NEE was measured between 1100 and 1600 hours at biweekly intervals throughout the 2010 and 2011 growing seasons. This frequency provided three to four midseason measurements on each of 70 plots. We report NEE values from the atmospheric perspective where negative values indicate net uptake of CO_2_ by the ecosystem, and positive values indicate a net release of CO_2_ to the atmosphere.

### Statistical analysis

We used Pearson’s correlation coefficients to examine the interrelationships in top and repeated cover between CTs as well as individual taxa. We tested for differences in LA_VP_ among PFTs and individual species across all plots using the non-parametric Kruskal-Wallis test, followed by Dunn’s post-hoc tests. To analyze the influence of the proportional cover of the CTs on plot-level NDVI we fitted linear multiple regression models with plot-level NDVI as the dependent variable and CT proportional cover as the independent variables; significant coefficients were interpreted as a CT’s influence (in units of NDVI) on the plot-level NDVI signal. The idea of using the multiple regression is that the plot-level NDVI value is a linear combination of the NDVI values of the constituent taxa weighted by their proportional cover. Next, to parse the spectral contributions of individual CTs we compared reflectance in the NIR and red bands and NDVI values for the monotypic plots using Kruskal-Wallis tests, followed by Dunn’s post-hoc tests. One-way ANOVAs followed by Tukey post-hoc tests were used to make the analogous spectral comparisons among the three dominant taxa. We examined the relationship between LA_VP_ or NDVI and peak season NEE_600_, GPP_600_, and *R*_*eco*_ using linear regression models.

Finally, we evaluated two models commonly used in the prediction of tundra productivity. The first, henceforth the “LAI~NDVI” model, has been used [10,11,37] to predict LAI from NDVI using the following relationship:

$$LAI=a*e^{b*NDVI}$$ (4)

We parameterized the curve using constrained nonlinear least squares estimation [38], with parameter bounds derived from Table 2 in Street [9] and the pan-Arctic parameters in Shaver (2013). Next, the Tundra GPP Model has often been used concurrently to estimate GPP using the modeled values of LAI:

$$GPP=-\left(\frac{A_{max}}{b}\right)*\ln\left[\frac{\left(-A_{max}+E_{0}*PAR\right)}{\left(-A_{max}+E_{0}*e^{-b*LAI}\right)}\right]$$ (5)

Where *b* is the Beer’s law extinction coefficient, set to 0.5 as in previous studies [11,39], and the remaining parameters are as previously defined. Models were evaluated by comparing our observed values against model predicted values (e.g., LAI modeled from NDVI compared to LA_VP_).

All analyses were carried out in R version 4.1.2 (R Core Team, 2021). Figures were produced using the R packages ggplot2 [40] and patchwork [41]. For multiple regression models, predictor variables were checked for collinearity, and for ANOVA and regression models all residuals were checked for normality and homogeneity of variance.

## Results

### Species cover

**Fig 1:**
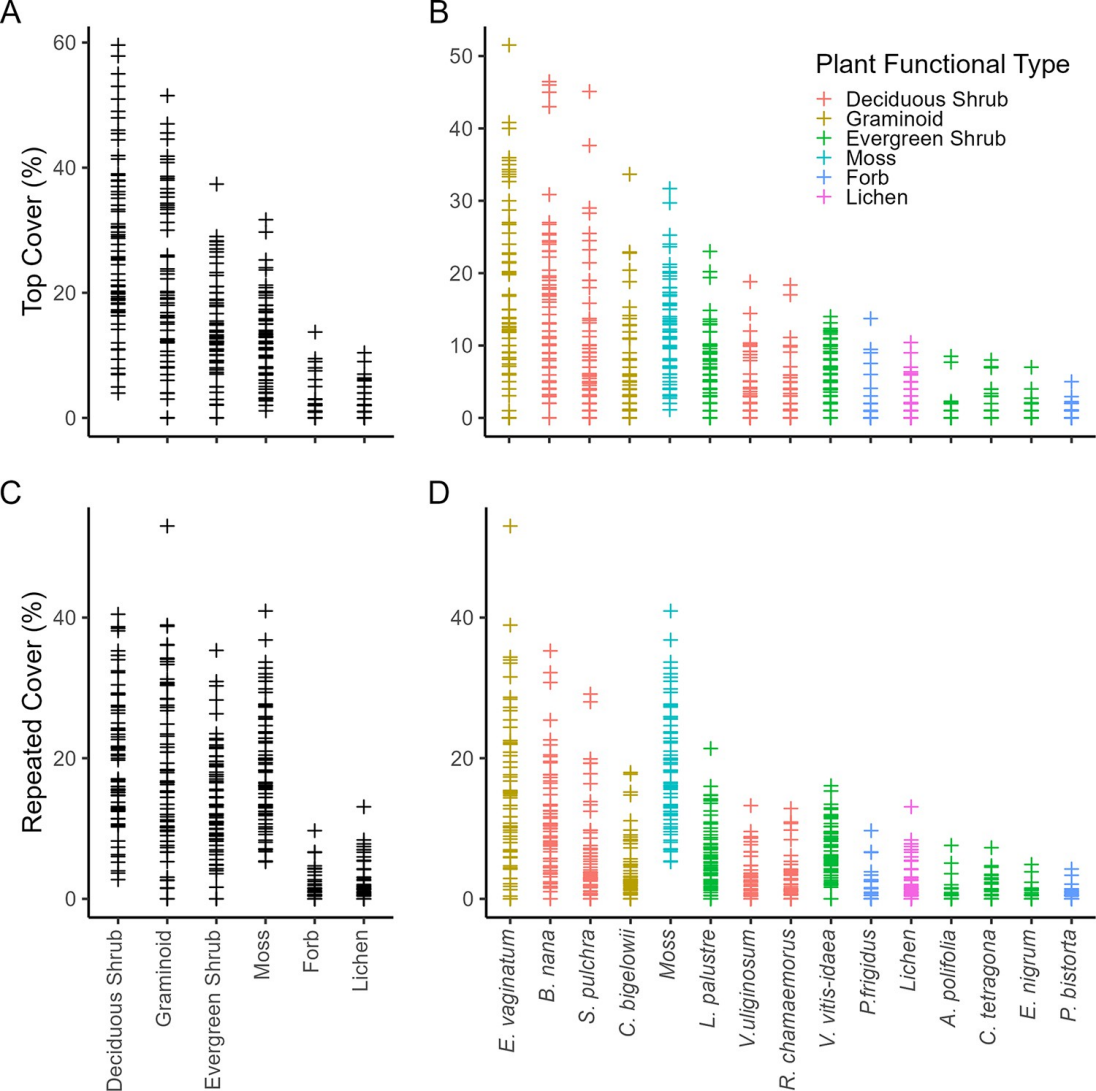
Sample plot vegetation top cover by plant functional type (A) and species (B), and repeated cover by plant functional type (C) and species (D) in the MAT sample plots. Points (plus symbols) are individual plots. Points in (B) and (D) are colored by species’ functional type. Moss and lichen were not identified to species.

As expected for MAT, deciduous shrubs had the highest average top cover followed by graminoids, but sample plots varied widely in the proportional contributions of deciduous shrubs and graminoids (Fig 1), satisfying the intent of the study. The mean ± standard error top cover of the deciduous shrub PFT, composed primarily of *B*. *nana* and *S*. *pulchra*, was 27.5 ± 1.6% and the mean ± standard error top cover of the graminoid PFT, composed primarily of *E*. *vaginatum* and *C*. *bigelowii*, was 21% ± 1.7%. Top cover of deciduous shrubs correlated strongly and inversely with graminoid top cover (*r* = -0.65, *P* = 0.04, n = 70, S4 Fig), primarily due to an inverse correlation between *S*. *pulchra* and *E*. *vaginatum* (*r* = -0.51, *P* = 0.001, n = 70, S5 Fig). Among taxa, *E*. *vaginatum* and *B*. *nana* were the most abundant canopy species based on top cover, surpassed by moss for total cover.

### Vertically projected leaf area

**Fig 2:**
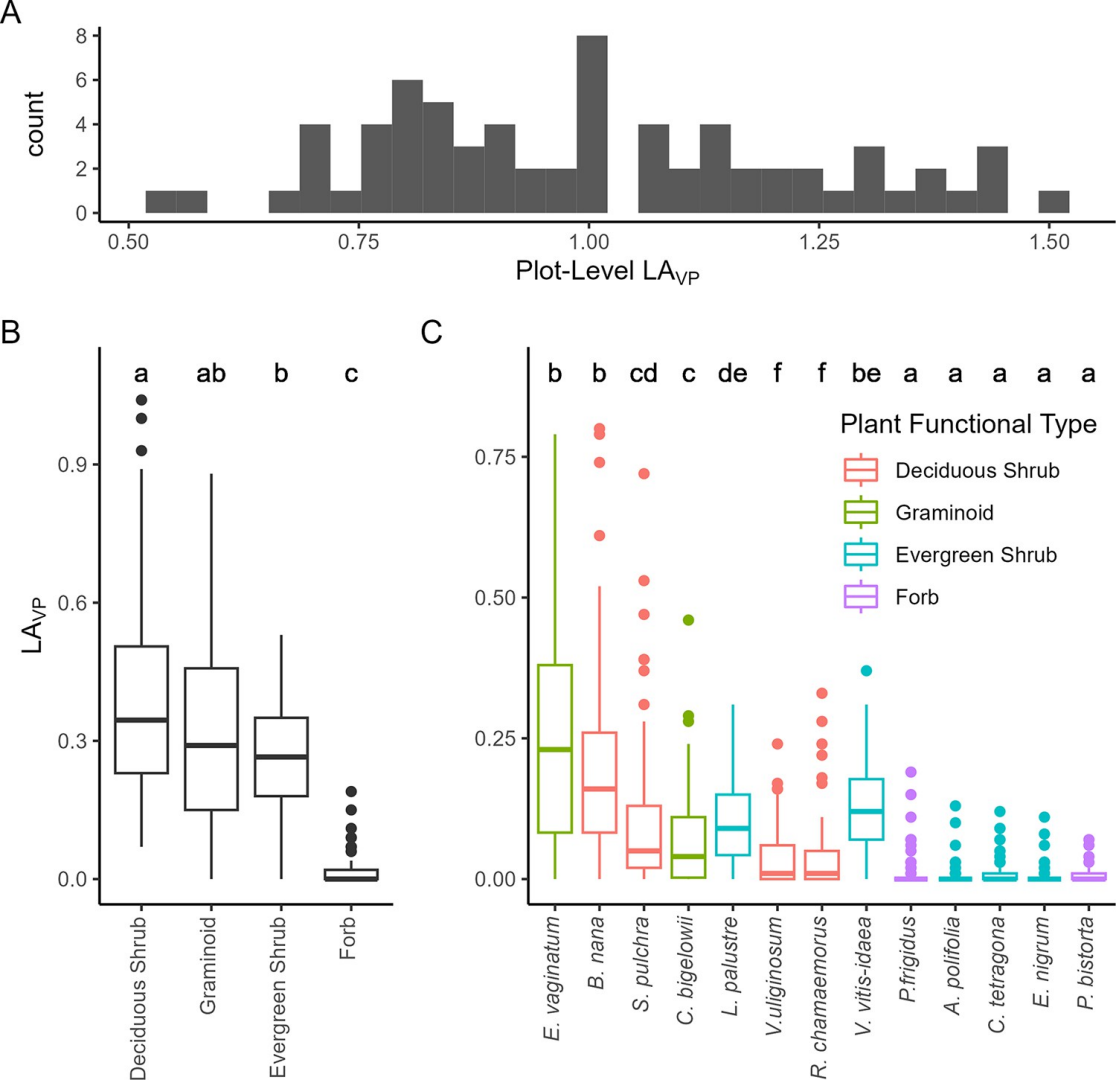
Plot-level LA_VP_ distribution in sample plots (A) and LA_VP_ for each PFT (B), and species (C) in the MAT sample plots. Boxplots in (C) are colored by species’ functional type assignment. Significant differences among PFTs or taxa (Dunn’s Test) in (B) and (C) are indicated by different letters.

Vertically projected leaf area (LA_VP_) varied from 0.54 to 1.51 with a mean value of 1.02 (Fig 2). The PFTs differed in plot-level LA_VP_ (Kruskal-Wallis test, *H*_3_ = 142, *P* < 0.001), due principally to differences in LA_VP_ between forbs and the pair deciduous shrubs and graminoids. Deciduous shrubs and graminoids exhibited the highest LA_VP_ (but did not differ significantly from each other (Fig 2B)). Individual taxa also differed in plot-level LA_VP_ (*H*_12_ = 447, *P* < 0.001), primarily due to differences between the pair *E*. *vaginatum* and *B*. *nana*, which did not differ significantly from each other, and several forb and evergreen dwarf shrub taxa.

### Spectral reflectance indices

**Fig 3:**
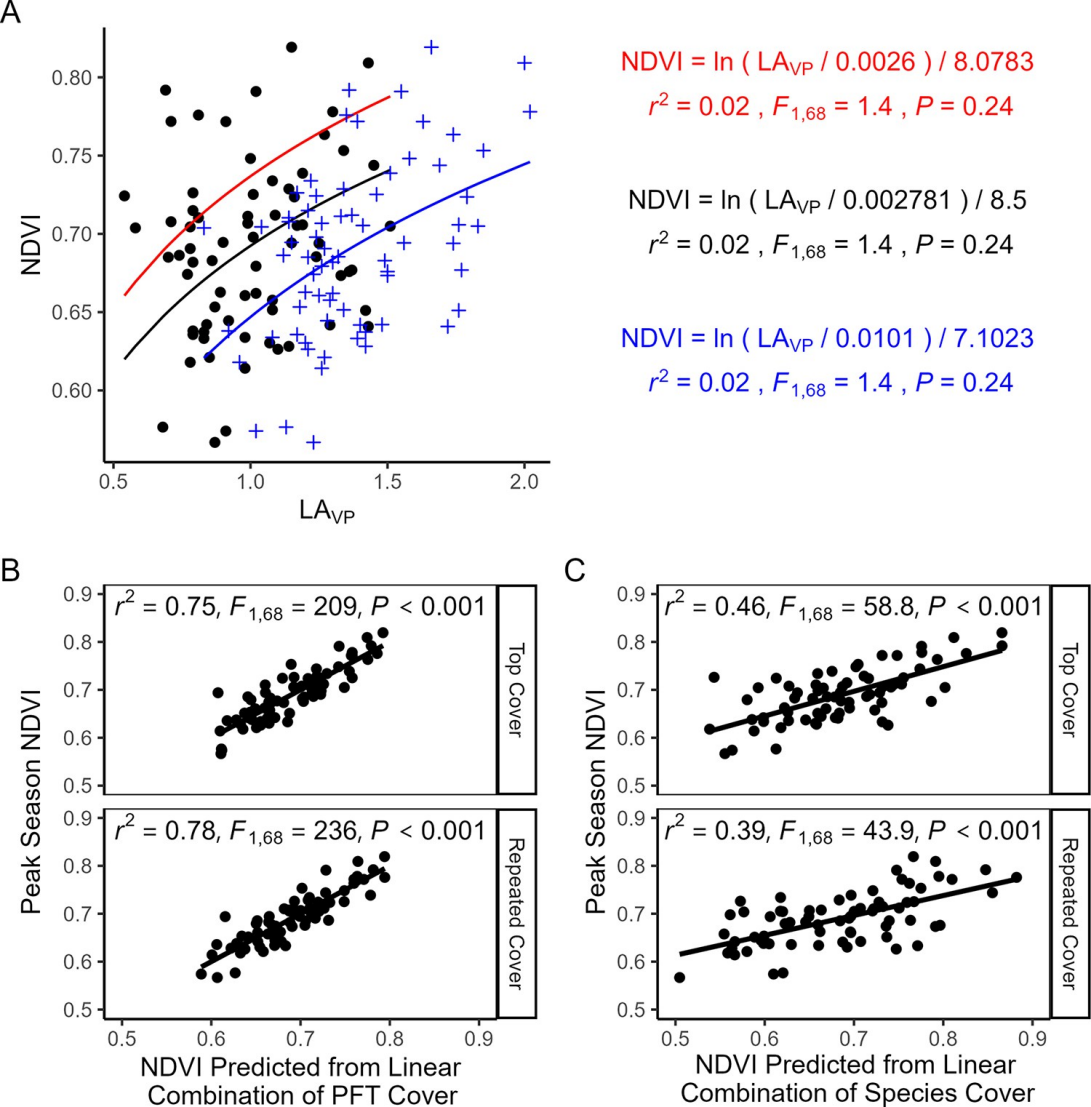
(A) Observed peak-season NDVI in the MAT sample plots regressed against plot-level vertically projected leaf area (LA_vp_) using the LAI~NDVI model (Eq 4), solved for NDVI. Black line and text show the best fit parameters estimated using nls with LA_VP_ calculated using vascular species only, red shows the pan-Arctic parameters used in Shaver (2007, 2013), and blue shows best fit parameters with LA_VP_ calculations that include moss. (B) Observed peak-season NDVI regressed against NDVI predicted from a multiple regression model using top cover (first pin hits only) or repeated cover of eight PFTs or (C) individual species as predictors. Goodness-of-fit statistics are inset. Regression coefficients are in Tables 1 and 2.

Peak season NDVI was not well predicted by LA_VP_ without moss included in LA_VP_ calculations (Fig 3) (*r*^*2*^ = 0.02, *F*_1,68_ = 1.4, *P* = 0.24). With moss included, LA_VP_ was a significant predictor of NDVI, but the fit was poor (*r*^*2*^ = 0.22, *F*_1,68_ = 18.7, *P* < 0.0001). Multiple regression models using top or repeated cover of the eight CTs to predict peak season NDVI values performed better, explaining 75 and 78% of the variation in NDVI (*r*^*2*^ = 0.72, *F*_8,61_ = 23.4, *P* < 0.001 and *r*^*2*^ = 0.75, *F*_8,61_ = 26.4, *P* < 0.001, respectively, Fig 3B). Regression coefficients for each CT except lichens were significantly greater than zero (P < 0.05, Table 1), with deciduous shrubs and forbs having the largest values (top cover regression coefficients = 0.91 and 1.28, respectively), followed by graminoids (top cover regression coefficient = 0.63). The individual species most influential in determining plot-level NDVI were *S*. *pulchra*, *B*. *nana*, and *V*. *uliginosum*, *Rubus chamaemorus*, *Empetrum nigrum*, and *Petasites frigidus* (Table 2).

**Table 1:** Summary of linear mixture models examining the influence of CT top cover (proportion of first pin hits) and repeated (proportion of all pin hits) cover on NDVI.

	PFT	estimate	se	*F*	*P*
Top Cover *F*_*5*,*64*_ = 23.4 *r*^*2*^ = 0.72 *P* < 0.0001	Forb	1.282	0.16	7.998	**<0.001**
	Deciduous Shrub	0.912	0.025	35.894	**<0.001**
	Litter	0.646	0.068	9.554	**<0.001**
	Evergreen Shrub	0.638	0.045	14.261	**<0.001**
	Graminoid	0.63	0.025	25.106	**<0.001**
	Dead	0.572	0.047	12.282	**<0.001**
	Moss	0.571	0.055	10.36	**<0.001**
	Lichen	0.262	0.179	1.467	0.147
Repeated Cover *F*_*5*,*64*_ = 26.4 *r*^*2*^ = 0.75 *P* < 0.0001	Forb	1.446	0.196	7.391	**<0.001**
	Deciduous Shrub	0.998	0.038	26.615	**<0.001**
	Litter	0.594	0.037	15.925	**<0.001**
	Evergreen Shrub	0.626	0.043	14.584	**<0.001**
	Graminoid	0.638	0.028	22.637	**<0.001**
	Dead	0.578	0.033	17.641	**<0.001**
	Moss	0.658	0.041	15.871	**<0.001**
	Lichen	0.264	0.144	1.837	0.071

*P* values in **bold** indicate significance at the *P* < 0.05 level.

**Table 2:** Summary of linear mixture models examining the influence of species top cover (proportion of first pin hits) and repeated (proportion of all pin hits) cover on NDVI.

	term	estimate	se	*F*	*P*
Top Cover *F*_*15*,*55*_ = 606 *r*^*2*^ = 0.99 *P* < 0.0001	*Petasites Frigidus*	2.088	0.34	6.138	**<0.001**
	*Salix pulchra*	1.266	0.116	10.947	**<0.001**
	*Ledum palustre*	1.063	0.177	6.012	**<0.001**
	*Betula nana*	1.003	0.069	14.589	**<0.001**
	*Vaccinium uliginosum*	0.975	0.2	4.868	**<0.001**
	*Rubus chamaemorus*	0.926	0.231	4.017	**<0.001**
	*Carex bigelowii*	0.825	0.122	6.747	**<0.001**
	*Empetrum nigrum*	0.776	0.766	1.014	0.315
	*Polygonum bistorta*	0.741	0.966	0.766	0.447
	Moss	0.739	0.119	6.216	**<0.001**
	*Vaccinium vitis-idaea*	0.709	0.227	3.122	**0.003**
	*Eriophorum vaginatum*	0.693	0.062	11.274	**<0.001**
	*Cassiope tetragona*	0.682	0.447	1.527	0.132
	Lichen	0.487	0.393	1.239	0.221
	*Andromeda polifolia*	-0.02	0.533	-0.037	0.97
Repeated Cover *F*_*5*,*64*_ = 412 *r*^*2*^ = 0.99 *P* < 0.0001	*Empetrum nigrum*	3.806	1.157	3.288	**0.002**
	*Petasites Frigidus*	1.996	0.63	3.168	**0.003**
	*Vaccinium uliginosum*	1.528	0.315	4.85	**<0.001**
	*Polygonum bistorta*	1.485	1.465	1.014	0.315
	*Salix pulchra*	1.34	0.206	6.491	**<0.001**
	*Rubus chamaemorus*	1.157	0.378	3.063	**0.003**
	*Betula nana*	1.153	0.126	9.128	**<0.001**
	Moss	0.901	0.116	7.769	**<0.001**
	*Carex bigelowii*	0.901	0.229	3.927	**<0.001**
	Lichen	0.806	0.432	1.868	0.067
	*Eriophorum vaginatum*	0.794	0.085	9.365	**<0.001**
	*Ledum palustre*	0.711	0.217	3.275	**0.002**
	*Vaccinium vitis-idaea*	0.659	0.268	2.462	**0.017**
	*Andromeda polifolia*	0.483	0.786	0.615	0.541
	*Cassiope tetragona*	-0.7	0.759	-0.922	0.361

*P* values in **bold** indicate significance at the *P* < 0.05 level.

**Fig 4:**
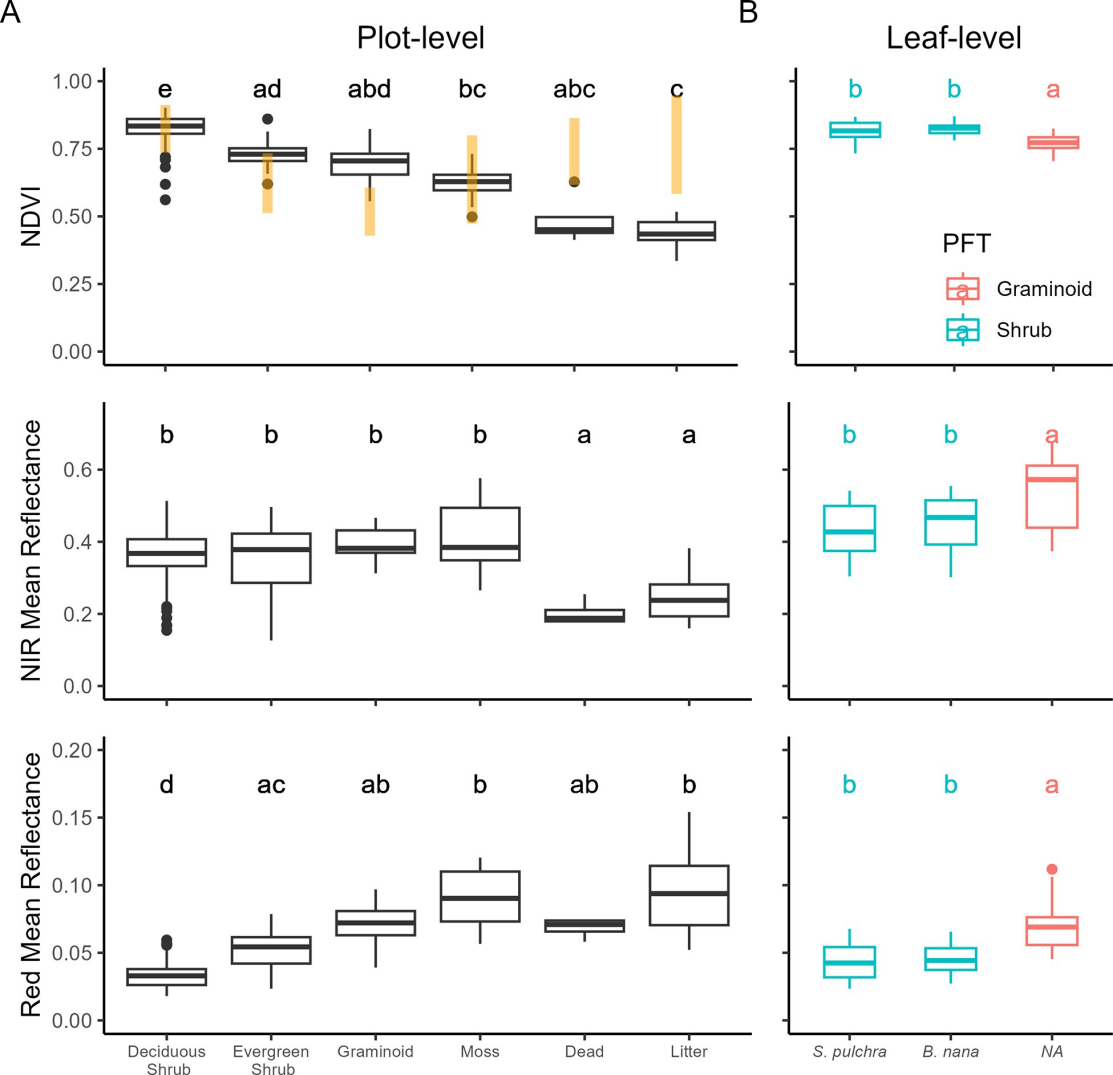
Reflectance signatures of CT-specific monotypic plots (A) and leaves from focal species (B). Significant differences among CTs or taxa (Dunn’s post-hoc test) in (A) and (B) are indicated by different letters. Orange bars in top left panel indicate regression coefficients (estimate ± se) from a multiple linear regression between CT top cover in the MAT sample plots and plot-level NDVI. The congruence between regression coefficients for the dominant CT’s and reflectance values from the monotypic plots supports our assertion that NDVI in mixed plots can be approximated as linear combinations of the product of CT top cover values.

The relative magnitude of regression coefficients for CTs corresponded with results from our monotypic patches (Fig 4A). *R*_*nir*_ differed among CTs (*H*_6_ = 57, *P* < 0.001), but was indistinguishable among the four leafy PFTs (graminoid, deciduous and evergreen shrubs, and moss). *R*_*red*_ also differed among CTs (*H*_6_ = 147, *P* < 0.001), but was significantly lower in deciduous shrub patches (0.033 ± 0.001) than in the other leafy PFTs (range: 0.053–0.92). As a result, NDVI was greatest for deciduous shrub patches (0.82 ± 0.01) compared to the other leafy PFTs (range: 0.63–0.73).

Spectral measurements displayed similar patterns at the leaf (Fig 4B) and monotypic levels. Mean *R*_*nir*_ differed among the three species tested (*F*_*2*,*86*_ *=* 13.9, *P* < 0.001), being higher for *E*. *vaginatum* leaves (0.68 ± 0.02) than for *B*. *nana* (0.53 ± 0.01) or *S*. *pulchra* leaves (0.54 ± 0.01). Likewise, reflectance in the visible range differed among species (*F*_*2*,*86*_ *=* 31.9, *P* < 0.001), being higher for *E*. *vaginatum* (0.074 ± 0.003) than for *B*. *nana* (0.057 ± 0.002) or *S*. *pulchra* leaves (0.63 ± 0.02). As a result, leaf-level NDVI differed among species (*F*_*2*,*86*_ *=* 25.3, *P* < 0.001), though the magnitudes of differences observed were small. *E*. *vaginatum* (0.76 ± 0.01) displayed lower leaf-level NDVI than the deciduous shrubs, *S*. *pulchra* (0.82 ± 05) and *B*. *nana* (0.82 ± 0.03).

### CO_2_ exchange

**Fig 5:**
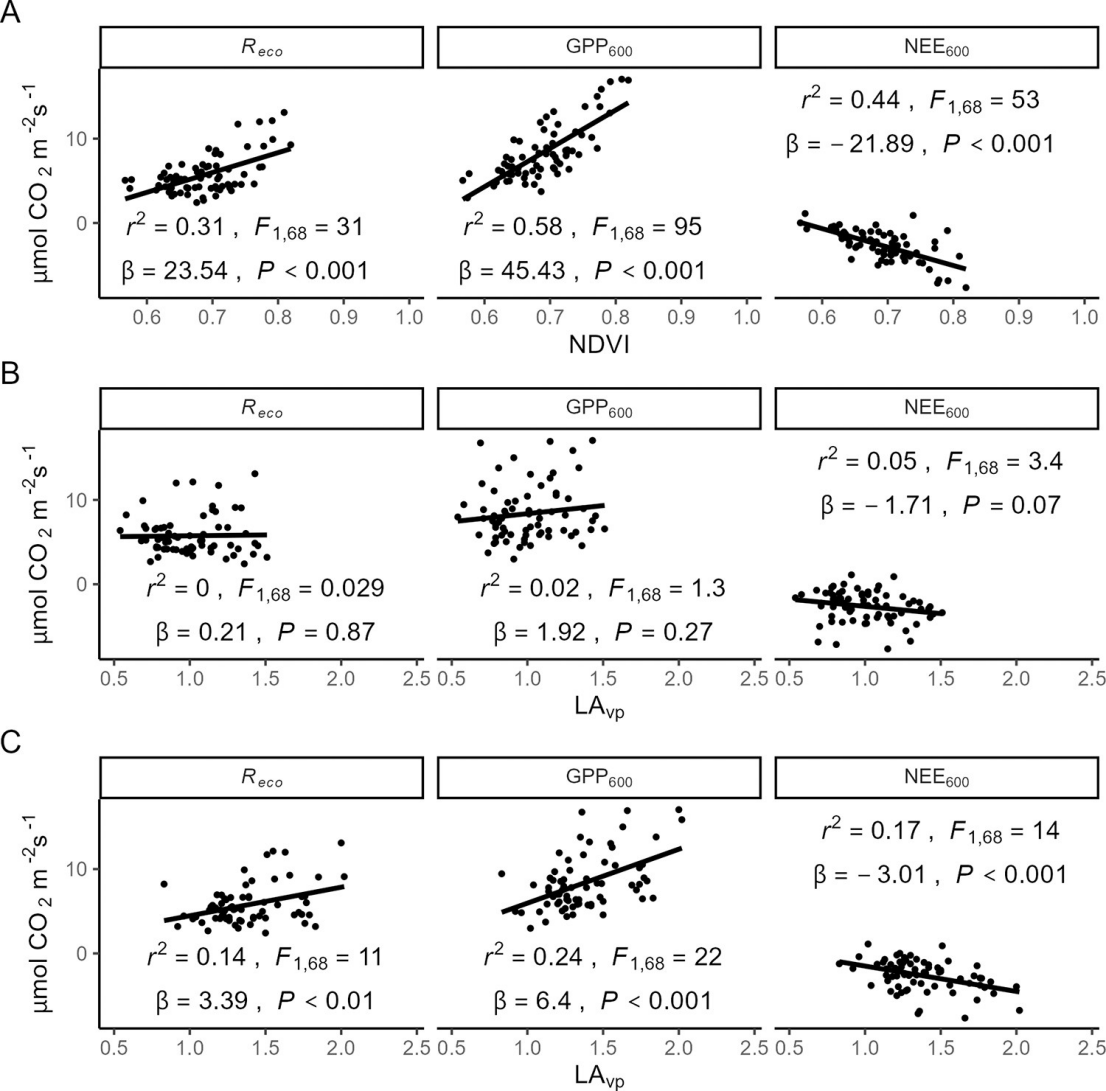
The relationship between components of peak-season plot-level CO_2_ exchange in the MAT sample plots and NDVI (A), LA_VP_ calculated without moss contributions (B), and LA_VP_ calculated with moss (C). Goodness-of-fit and model statistics are inset.

NDVI was a significant predictor of peak season plot-level CO_2_ exchange, being negatively correlated with NEE_600_ (increasing CO_2_ uptake associated with increasing NDVI) and positively correlated with GPP_600_ and *R*_*eco*_ (Fig 5A). We found no evidence of a direct relationship between plot-level LA_VP_ and NEE_600_, GPP_600_, or *R*_*eco*_ unless moss was included in LA_VP_ calculations (Fig 5B and 5C). With moss included, LA_VP_ was a significant predictor of NEE_600_, GPP_600_, and *R*_*eco*_, but the fit was poor (*r*^2^ < 0.25 for all three models).

Regression models using top cover of the five photosynthetic PFTs to predict peak season NEE_600_, GPP_600_, and *R*_*eco*_ were significant (*P* < 0.001 for all three) and fit the data somewhat better, explaining 39%, 48%, and 22% of the variation, respectively (Table 3). Deciduous shrub and forb cover were negatively correlated with NEE_600_ and positively correlated with GPP_600_ (increasing CO_2_ uptake associated with increasing shrub and forb cover), while moss also exhibited a positive effect on GPP_600_. Forb cover was also positively correlated with *R*_*eco*_, being the only PFT to exhibit a significant relationship with this variable.

**Table 3:** Summary of regression models examining the influence of plant functional type top cover (proportion of first pin hits) on plot-level CO_2_ exchange.

response	term	estimate	SE	*F*	*P*
NEE_600_ *F*_*5*,*64*_ = 9.18 *r*^*2*^ = 0.37 *P* < 0.0001	Deciduous Shrub	-10.042	2.458	-4.086	**<0.001**
	Evergreen Shrub	0.208	2.96	0.07	0.944
	Graminoid	-4.783	2.547	-1.878	0.065
	Moss	-3.46	3.03	-1.142	0.258
	Forb	-19.002	8.01	-2.372	**0.021**
	Lichen	6.576	8.595	0.765	0.447
GPP_600_ *F*_*5*,*64*_ = 12.0 *r*^*2*^ = 0.44 *P* < 0.001	Deciduous Shrub	16.209	4.191	3.868	**<0.001**
	Evergreen Shrub	-0.909	5.048	-0.18	0.858
	Graminoid	5.778	4.342	1.331	0.188
	Moss	10.383	5.167	2.01	**0.049**
	Forb	43.237	13.658	3.166	**0.002**
	Lichen	1.028	14.656	0.07	0.944
*R*_*eco*_ *F*_*5*,*64*_ = 4.9 *r*^*2*^ = 0.22 *P* < 0.001	Deciduous Shrub	6.167	3.486	1.769	0.082
	Evergreen Shrub	-0.701	4.198	-0.167	0.868
	Graminoid	0.994	3.612	0.275	0.784
	Moss	6.923	4.297	1.611	0.112
	Forb	24.234	11.36	2.133	**0.037**
	Lichen	7.604	12.19	0.624	0.535

*P* values in **bold** indicate significance at the *P* < 0.05 level. Model fits are displayed under the response variable.

Plot-level light curve parameters were partially predicted by PFT cover (Table 4). The initial slope of the light curve, *E*_*0*_, was significantly predicted by cover of the five photosynthetic PFTs (*F*_*5*,*62*_ = 10.6, *r*^*2*^ = 0.46, *P* < 0.001), with deciduous shrubs and forbs driving steeper initial slopes (a more rapid increase in ecosystem CO_2_ uptake in response to increasing light). The LCP (where GPP = *R*_*eco*_) was significantly predicted by cover of the five photosynthetic PFTs, but the fit was poor (*F*_*5*,*62*_ = 2.7, *r*^*2*^ = 0.18, *P* = 0.03). Nonetheless, deciduous shrub cover displayed a significant negative relationship with the LCP.

**Table 4:** Summary of regression models examining the influence of plot top cover (proportion of first pin hits) on plot-level light curve parameters.

response	term	estimate	SE	*F*	*P*
*E*_*0*_ *F*_*5*,*62*_ = 11.7 *r*^*2*^ = 0.49 *P* < 0.0001	Deciduous Shrub	-0.027	0.007	-3.774	**<0.001**
	Evergreen Shrub	0.001	0.009	0.082	0.935
	Graminoid	-0.007	0.008	-0.903	0.37
	Moss	-0.017	0.01	-1.74	0.087
	Forb	-0.077	0.024	-3.189	**0.002**
LCP *F*_*5*,*62*_ = 3.03 *r*^*2*^ = 0.19 *P* = 0.02	Deciduous Shrub	-651.227	245.091	-2.657	**0.01**
	Evergreen Shrub	-192.741	310.636	-0.62	0.537
	Graminoid	-271.743	261.974	-1.037	0.304
	Moss	405.787	323.409	1.255	0.214
	Forb	-780.796	815.605	-0.957	0.342

*P* values in **bold** indicate significance at the *P* < 0.05 level. Model fit statistics are displayed under the response variable. *E*_*0*_ *=* slope of the light curve at PAR = 0. LCP = Light Compensation Point, where GPP and ER are balanced, and NEE = 0. We report all CO_2_ exchange data from the atmospheric perspective (more negative NEE values indicate increased CO_2_ uptake); this is reflected in the coefficients below (i.e., a lower coefficient in the *E*_0_ model indicates a higher quantum yield).

### Model evaluation

**Fig 6:**
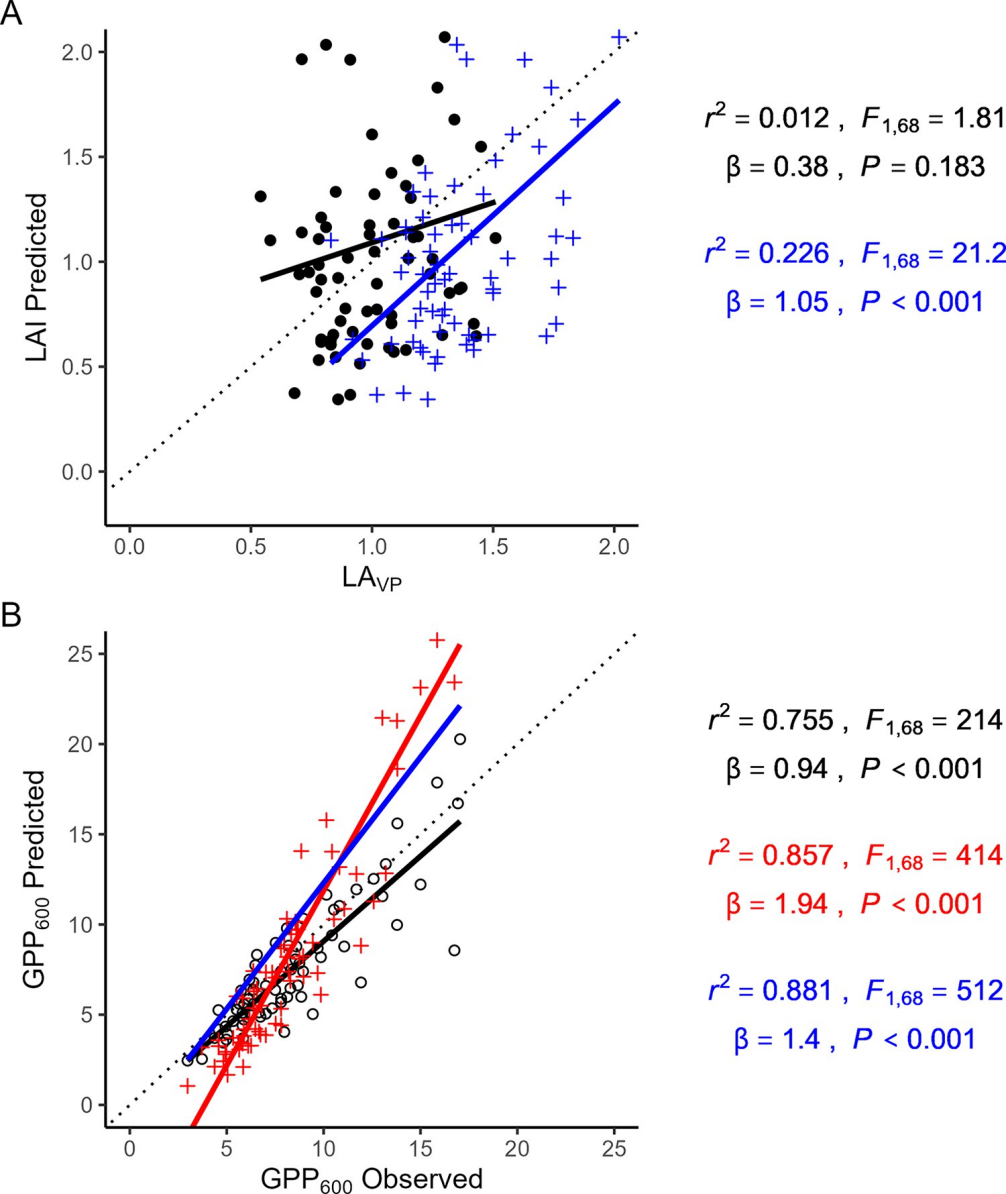
(A) Relationship between LAI predicted using the LAI~NDVI model parameterized with field plot data and observed LA_VP_. Black points and lines show the fit with LA_VP_ calculated without moss included, whereas the blue line shows the fit with LA_VP_ calculated *with* moss contributions. (B) Relationship between GPP_600_ predicted by the Tundra GPP Model (Eq(5)) using LA_VP_ without moss (black), LA_VP_ with moss (blue), or LAI modeled from NDVI (red) and GPP_600_ estimated from field measured light curves. In all plots, the dotted line represents a 1:1 relationship.

Without moss included in LA_VP_ calculations the LAI ~ NDVI Model (Eq (4)) did not predict observed LA_VP_ well (*r*^2^ = 0.012, *P* = 0.18, Fig 6A), overpredicting lower LA_VP_ values and underpredicting higher values (predicted vs. observed slope = 0.38). With moss included in LA_VP_, the model performed better but the fit was still poor (*r*^2^ = 0.23, *P* < 0.001, predicted vs. observed slope = 1.05) As a result, predictions from the Tundra GPP Model (Eq (5)) using LAI modeled from NDVI correlated with observed values (*r*^2^ = 0.77, *P* < 0.001, Fig 6B), but did not follow a 1:1 relationship, tending to overestimate higher rates of GPP_600_ and underestimate lower rates (predicted vs. observed slope = 1.94). When LA_VP_ was used in the Tundra GPP Model, predicted values followed observed values very closely (*r*^2^ = 0.76, predicted vs. observed slope = 0.94, *P* < 0.001).

## Discussion

Our multi-scale investigation set out to answer two questions regarding NDVI, plant structure, and ecosystem function. First, does increasing NDVI in MAT indicate an increase in leaf area, deciduous shrub abundance, or both? And second, what are the implications of observed variation in NDVI for NEE, GPP, and ecosystem respiration (*R*_*eco*_)?

In answer to our first question, we found that NDVI is dominated by the biochemical characteristics of the leaves of the primary PFTs in MAT, more so than by cumulative leaf area (i.e. LA_VP_). In the MAT plots, NDVI was more strongly driven by top cover and repeated cover of deciduous shrubs than any other co-dominant PFTs, and deciduous shrub monotypic plots exhibited much higher NDVI than other PFTs. Surprisingly, in our study plot-level vascular LA_VP_ (LAI typically only accounts for vascular vegetation [9]) was a poor predictor of NDVI. In answer to our second question, we found that vascular LA_VP_ was a poor direct predictor of NEE, GPP, and *R*_*eco*_, whereas NDVI or PFT cover were significant predictors of all three fluxes. Together, these lines of evidence support the interpretation of recent increases in NDVI within MAT of the North American Arctic as indicative of increasing deciduous shrub abundance [16,18,42,43], with substantial implications for C uptake [44], forage quality [45], and ecosystem hydrology [46].

Our multiple regression results suggest that midsummer NDVI in MAT is effectively a two-dimensional measurement of vegetation that is determined by the spectral characteristics of leaves at the top of the canopy. Despite making contributions to plot-level LA_VP_ that were statistically similar to those of graminoids (Fig 2), the regression coefficients demonstrate that deciduous shrubs dominate the NDVI signal in this ecosystem (Table 1), as has been noted in other tundra studies [22,47]. This is supported by the two dominant deciduous shrubs, *B*. *nana* and *S*. *pulchra*, exhibiting higher leaf-level NDVI than the dominant graminoid, *E*. *vaginatum*, primarily due to much lower reflectance in the red band (Fig 4B), which was consistent with the patterns we observed when comparing the pure canopies of the monotypic plots (Fig 4A). Thus, areas exhibiting increasing NDVI values within MAT [29] likely indicate horizontally expanding deciduous shrub canopies [48].

Next, many tundra-focused studies have used NDVI to estimate leaf area index (LAI) [9,11,49]. Thus, we were surprised to find no relationship in our data between NDVI and whole plot LA_VP_ (Fig 3), which was further reflected in the poor fit between LAI modeled from NDVI (Eq (4)) and LA_VP_ (Fig 6). Including moss in the LA_VP_ calculation changed the LAI~NDVI relationship to statistically significant as LA_VP_ captured more photosynthetically active tissues, but the fit between observed LA_VP_ and LAI predicted from NDVI was still poor. Although LA_VP_ is a variant that underestimates LAI [47], this metric (based on a point-frame with vertical pins) has been found to be closely related to LAI and aboveground biomass in other non-forested ecosystems [33,50]. Further, use of LA_VP_ in the Tundra GPP Model yielded excellent agreement with observed levels of GPP (Fig 6), further validating it as a non-destructive estimator of LAI in this system. One explanation for the poor fit between LAI and NDVI may lay in high variation in leaf angle [47,51] and biochemical characteristics [45,52,53] in tussock tundra communities; both affect the reflectance characteristics of leaves when viewed from above (as in a NDVI measurement). However, this does not explain the better fits between LAI and NDVI found in previous studies [9,12]. This raises the possibility that variation in LAI and variation in deciduous shrub abundance may have been confounded in previous studies [9–12]. Plots with high LAI and high NDVI values may have exhibited high NDVI values because of abundant deciduous shrubs rather than because of high LAI alone. The implication of this finding is that NDVI may be a better tool for examining spatiotemporal variation in deciduous shrub abundance in Arctic MAT systems than previously thought.

While NDVI was not closely correlated with LA_VP_, it was closely correlated with GPP and NEE (Fig 5). Meanwhile, there was no evidence of a correlation between LA_VP_ and GPP or NEE, despite LAI being one of the most commonly used predictors of CO_2_ exchange in Arctic tundra [10,11]. Again, including moss in the LA_VP_ calculations changed these model fits to statistically significant, but the relationship was still relatively coarse. Some of this may be due to our measurements of plot-level photosynthesis occurring during peak light conditions (a six-hour window straddling solar noon); the more vertically oriented leaves of graminoids [47,51] (which contribute substantially to LAI in this system (Fig 2B)) may demonstrate greater photosynthesis (relative to shrubs) with the sun at a lower angle. On the other hand, using a six-hour window and two months of the growing season we may have captured enough variation in solar angle to accurately represent the contributions of graminoids. In this case, results from our regression that used PFT cover to predict CO_2_ exchange (Table 3) indicate that greater *in situ* photosynthesis by deciduous shrub canopies leads to the strong correlation between NDVI and CO_2_ exchange.

Of the dominant PFT’s, deciduous shrubs had the only significant relationship with both NEE and GPP. Analysis of the light curves suggest this is partly due to the higher quantum yield of deciduous shrub-dominated plots (Table 4) as well as the negative correlation between deciduous shrub cover and the plot-level LCP; as a result, deciduous shrub-dominated plots will achieve net uptake at lower light levels than plots dominated by other PFTs. Recent work with MAT species corroborates these patterns; *S*. *pulchra* in particular tends to demonstrate higher light-saturated photosynthesis than *E*. *vaginatum* during mid to late summer [52,54], while *B*. *nana* has been found in some cases to exhibit greater photosynthesis throughout the season than *E*. *vaginatum* [55].

These findings are important in two ways. First, as vegetation in the Arctic change continues to change [26], these differences in leaf-level physiology between deciduous shrubs and graminoids in MAT will have substantial consequences for ecosystem C budgets. Second, studies that intend to model CO_2_ exchange in the Arctic using remotely sensed spectral indices may benefit from considering NDVI as an indirect predictor of CO_2_ exchange through its relationship with deciduous shrub abundance (and thus canopy photosynthetic capacity), with LAI as an independently estimated and potentially complimentary predictor. In our dataset, using NDVI to estimate LAI and LAI, in turn, to estimate CO_2_ exchange [9–12] yielded GPP estimates that were substantially out of step with observed values (Fig 6B).

Amplified warming and changing spectral signatures of Arctic landscapes highlight the need for ground truthing efforts that link spectral reflectance with ecosystem structure and function. Our dataset, collected in one of the most spatially extensive vegetation communities in the Arctic, is consistent with an interpretation of Arctic greening as a phenomenon of deciduous shrubs expanding both horizontally and vertically, overtopping graminoids and thereby increasing NDVI [18,26] and C uptake [49]. Moreover, it appears that reflectance values at the plot scale can be approximated as linear combinations of the product of PFT top cover values, with shrub (and forb) cover the strongest drivers of the NDVI signal. On the other hand, NDVI displays little relationship with LA_VP_ in moist acidic tundra, as leaf biochemical traits may override leaf quantity in both spectral and functional significance.

## Supporting information

S1 Fig A leaf-level measuring device was constructed and used with a Unispec-DC to scan individual leaves in the field. (TIF)

S2 Fig Relationship between model (nlme) predicted *R*_*eco*_ and “observed” *R*_*eco*_ as estimated by a full shade NEE measurement. OLS fit statistics: slope = 0.92, *r*^*2*^ = 0.92, *P* < 0.0001.
(TIF)

S3 Fig (A) Relationship between fitted values for the half-saturation constant (*k*) and light-saturated photosynthetic rate (*A*_*max*_). (B) and (C) display the relationship between *A*_*max*_, *k*, and day of year, respectively. Values are from all light curves fit in the study (n = 404).
(TIF)

S4 Fig Correlation matrices displaying strength and direction of correlation for cover proportions among the different plant functional types present in top cover (A) and repeated cover (B). An “X” represents a statistically significant correlation at *P* < 0.05 level.
(TIF)

S5 Fig Correlation matrices displaying strength and direction of correlation for cover proportions among the different species present in top cover (A) and repeated cover (B). An “X” represents a statistically significant correlation at *P* < 0.05 level.
(TIF)
