# Supplementary figures and images for: NDVI changes in the Arctic: Functional significance in the moist acidic tundra of Northern Alaska
Source: PLoS One. 2023 Apr 28;18(4):e0285030. doi: 10.1371/journal.pone.0285030 (PMC10146450; doi:10.1371/journal.pone.0285030)

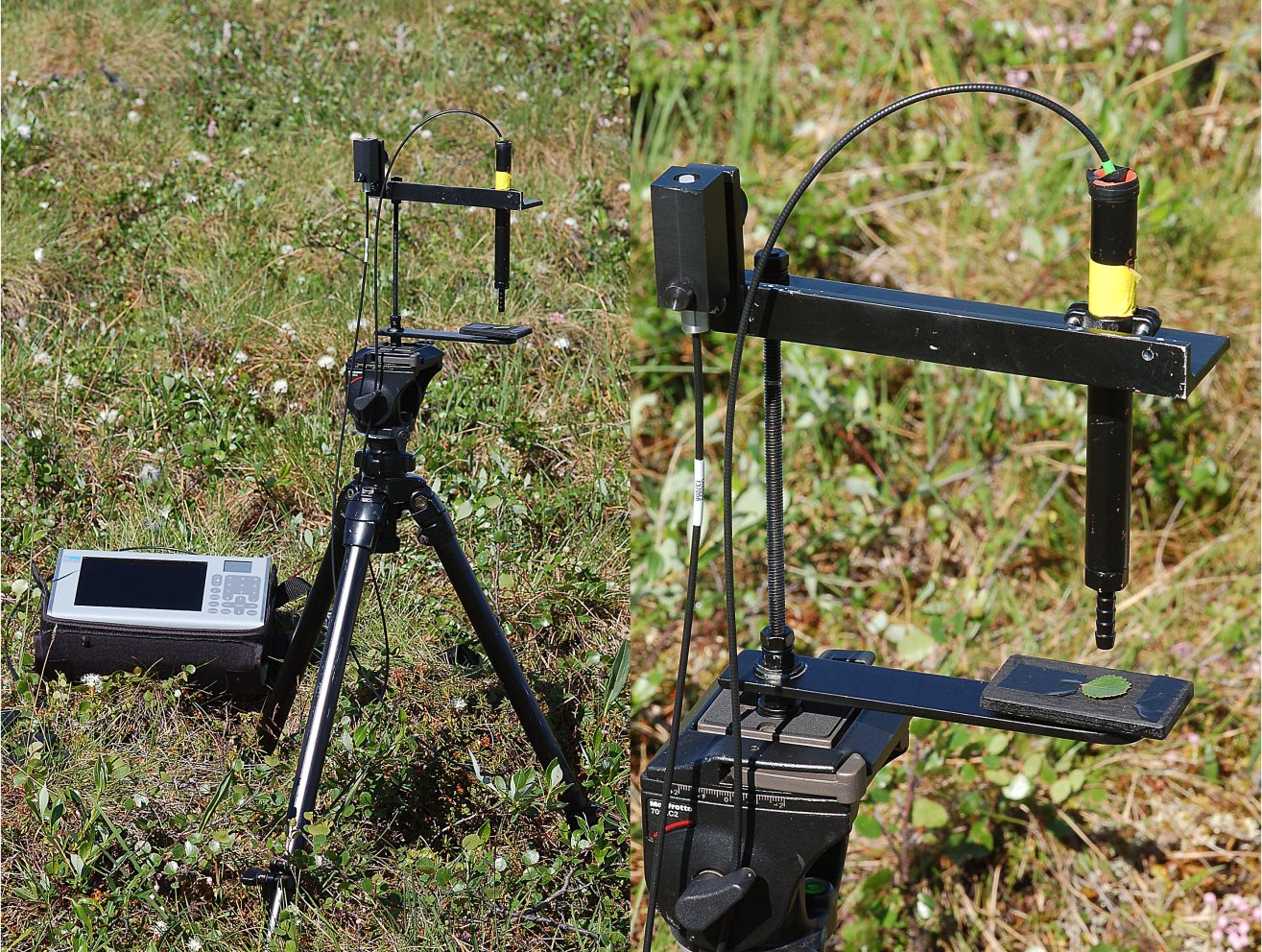

Supplement: S1 Fig — (TIF) [file pone.0285030.s001.tif]

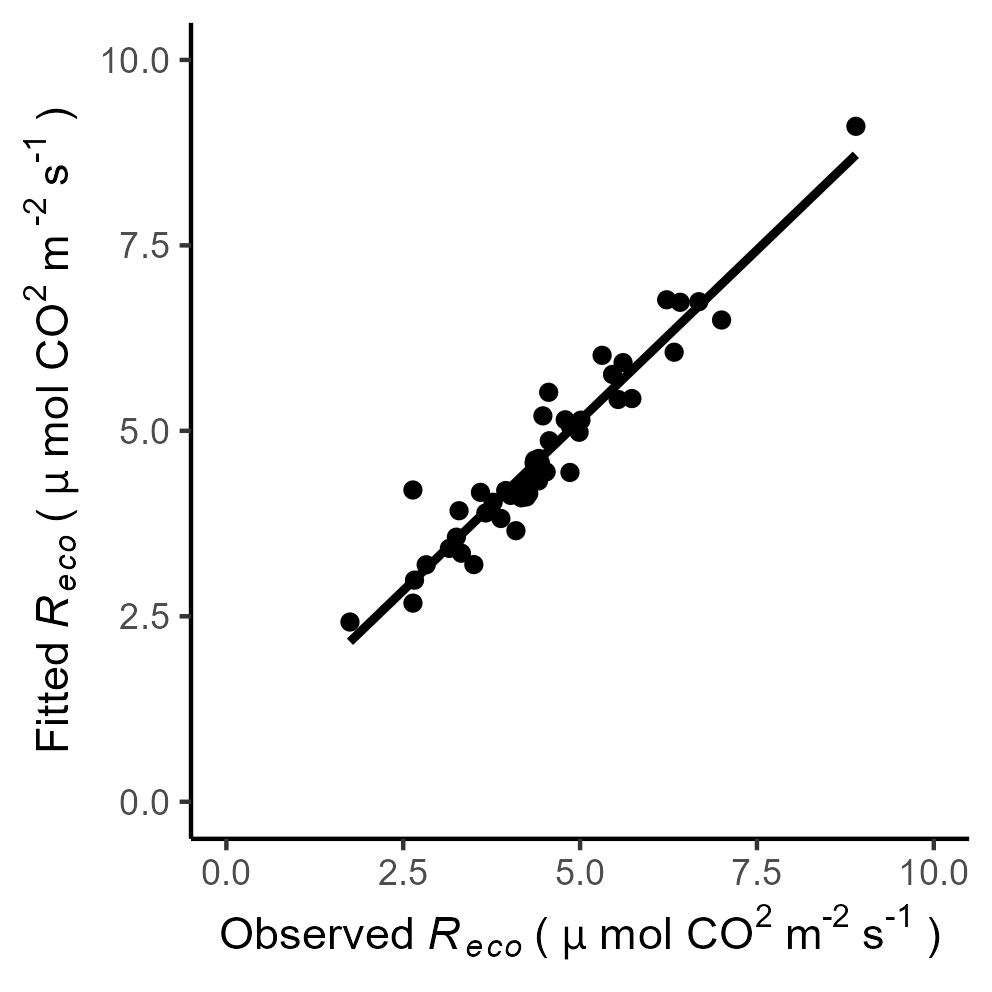

Supplement: S2 Fig — OLS fit statistics: slope = 0.92, r2 = 0.92, P < 0.0001. (TIF) [file pone.0285030.s002.tif]

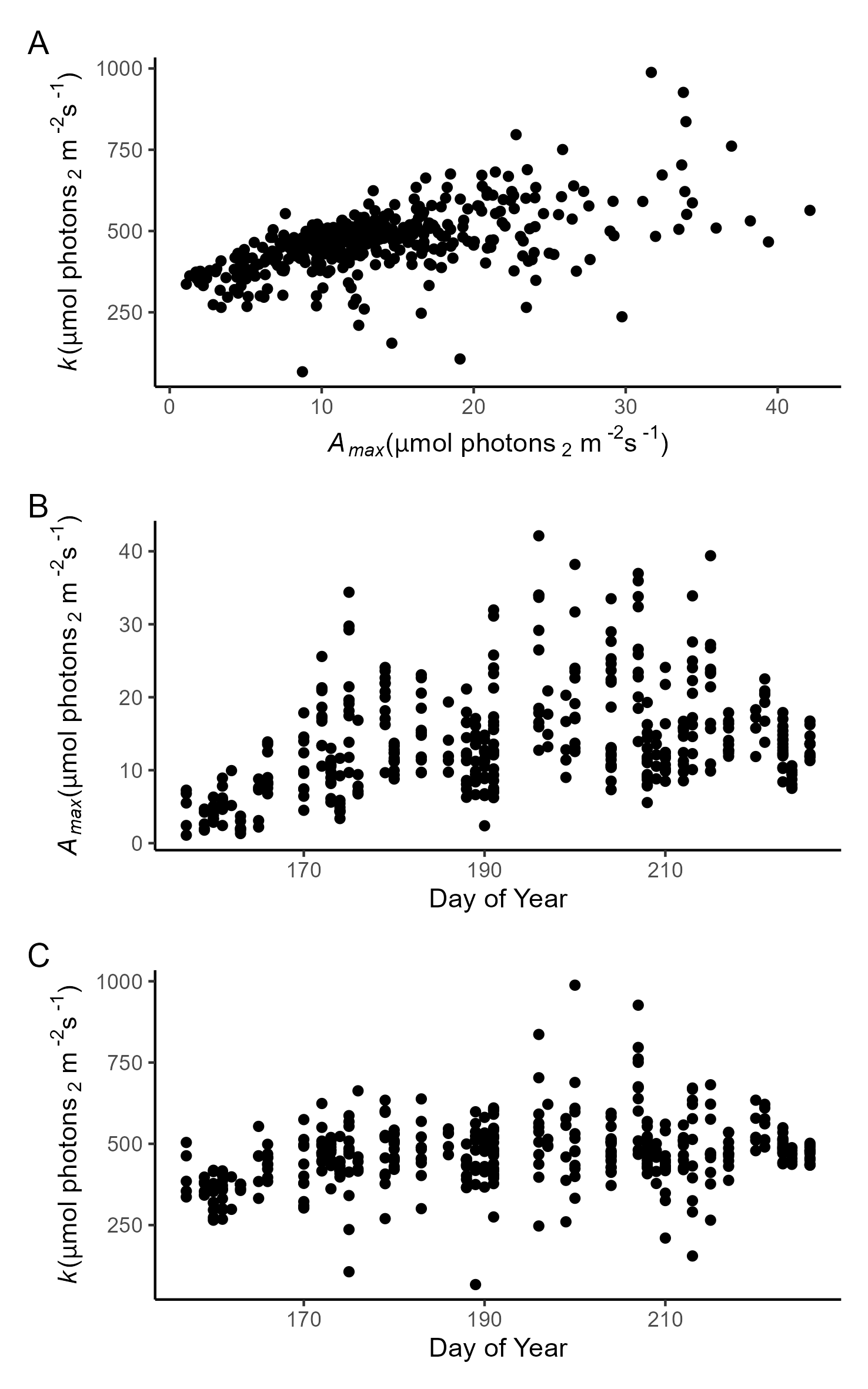

Supplement: S3 Fig — (A) Relationship between fitted values for the half-saturation constant (k) and light-saturated photosynthetic rate (Amax). (B) and (C) display the relationship between Amax, k, and day of year, respectively. Values are from all light curves fit in the study (n = 404). (TIF) [file pone.0285030.s003.tif]

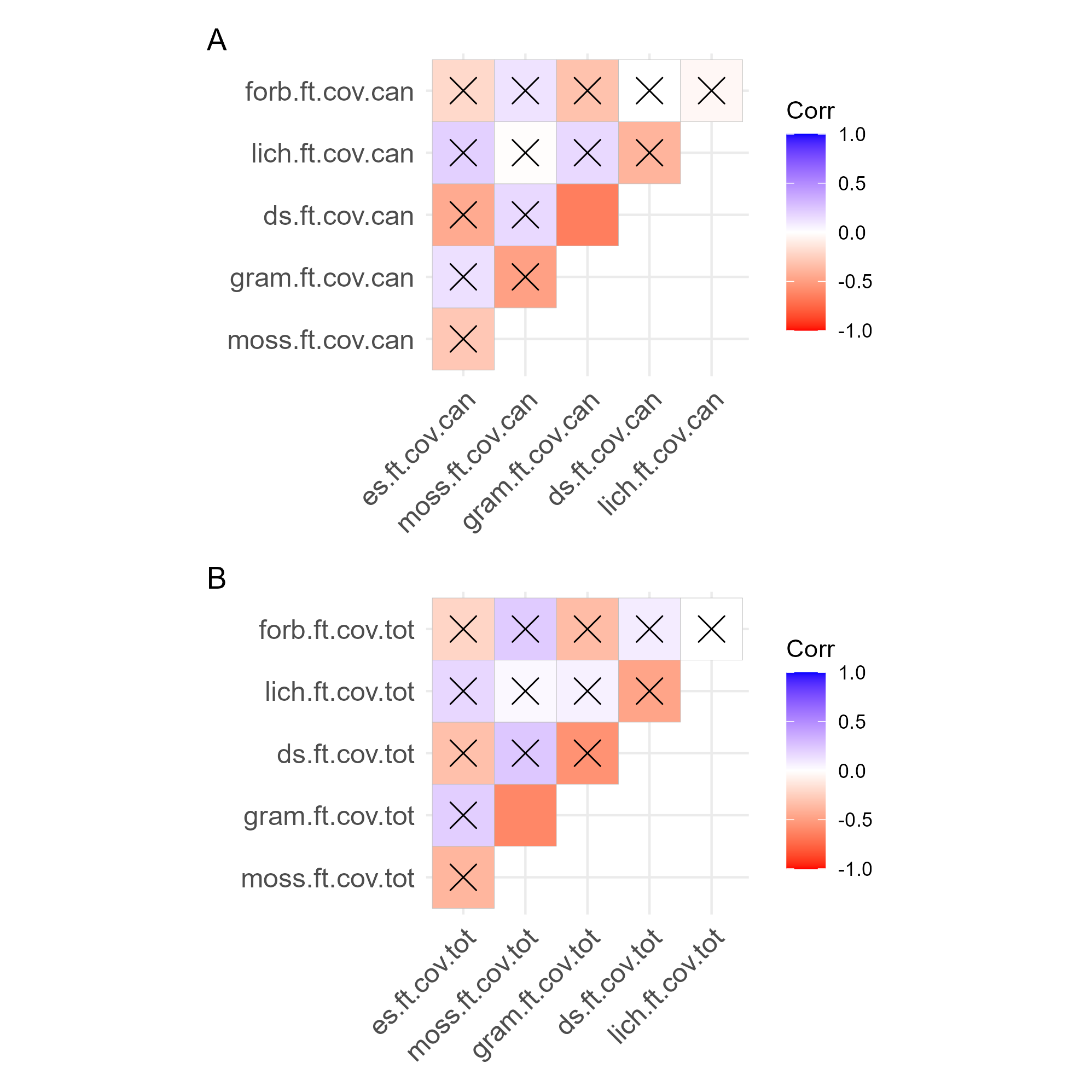

Supplement: S4 Fig — Correlation matrices displaying strength and direction of correlation for cover proportions among the different plant functional types present in top cover (A) and repeated cover (B). An “X” represents a statistically significant correlation at P < 0.05 level. (TIF) [file pone.0285030.s004.tif]

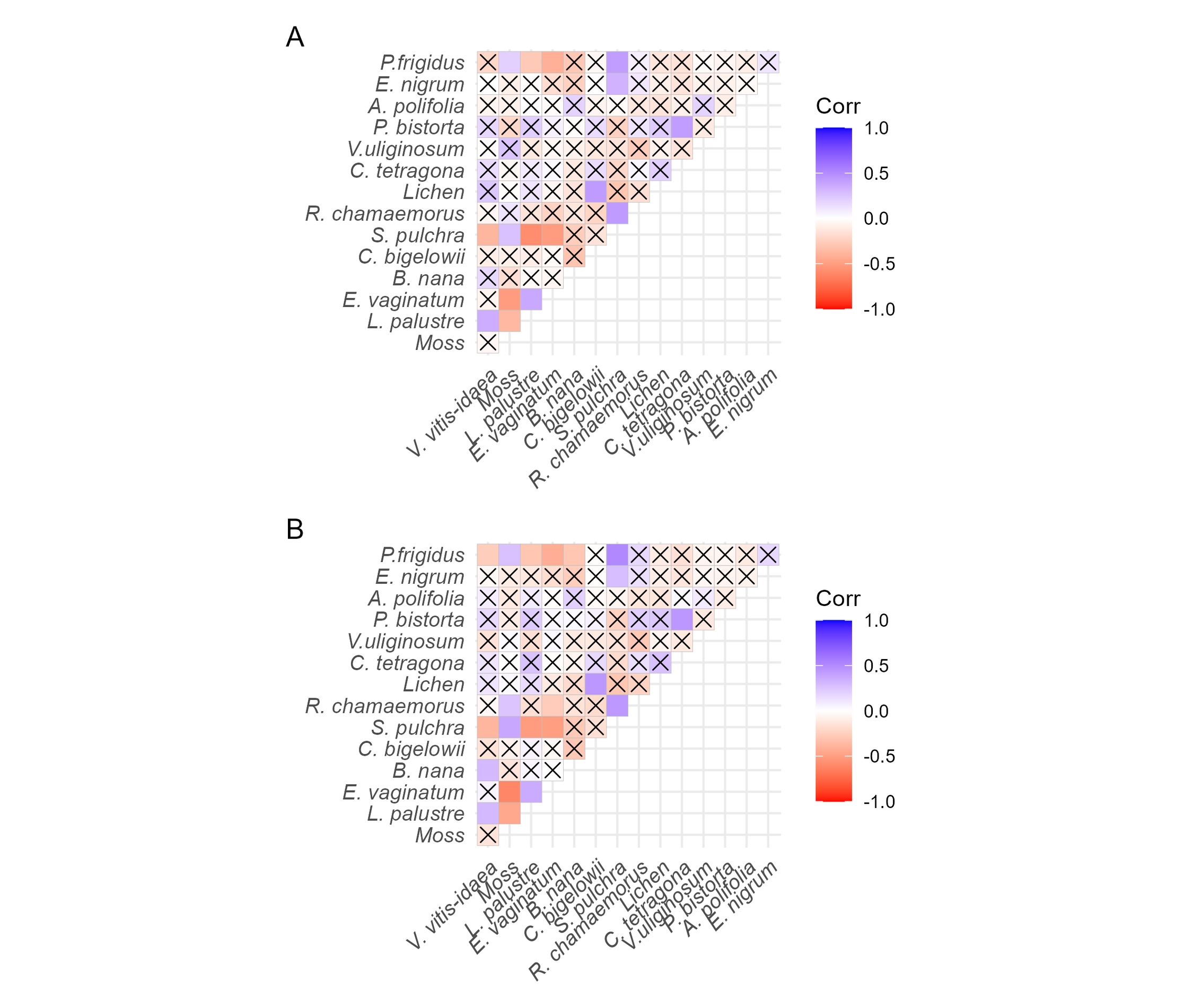

Supplement: S5 Fig — Correlation matrices displaying strength and direction of correlation for cover proportions among the different species present in top cover (A) and repeated cover (B). An “X” represents a statistically significant correlation at P < 0.05 level. (TIF) [file pone.0285030.s005.tif]
